# Characteristics of *A20* gene polymorphisms and clinical significance in patients with rheumatoid arthritis

**DOI:** 10.1186/s12967-015-0566-1

**Published:** 2015-07-05

**Authors:** Lihua Zhu, Liang Wang, Xu Wang, Lingling Zhou, Ziwei Liao, Ling Xu, Huixia Wu, Jie Ren, Zhaoxia Li, Lijian Yang, Shaohua Chen, Bo Li, Xiuli Wu, Yi Zhou, Yangqiu Li

**Affiliations:** Department of Rheumatism and Immunology, First Affiliated Hospital, Jinan University, Guangzhou, 510632 China; Institute of Hematology, Jinan University, Guangzhou, 510632 China; Department of Oncology, First Affiliated Hospital, Jinan University, Guangzhou, 510632 China; Key Laboratory for Regenerative Medicine of Ministry of Education, Jinan University, Guangzhou, 510632 China

**Keywords:** *A20* gene, Polymorphism, Mutation, Rheumatoid arthritis

## Abstract

**Background:**

There are a number of studies regarding to the susceptibility of *A20* SNPs in rheumatoid arthritis (RA); however, a few of these studies have shown an association between polymorphisms in the *A20* gene and RA risk in the Chinese population. The aim of this study was to investigate the characteristics of *A20* gene polymorphisms, the association between polymorphisms and clinical significance in Chinese RA patients.

**Methods:**

PCR and sequencing were used to identify *A20* gene polymorphisms in peripheral blood mononuclear cells (PBMCs) (50 cases), synovial fluid (11 cases) from RA patients and PBMCs from 30 healthy individuals. Quantitative Real-time PCR (qRT-PCR) was used to analyze the *A20* mRNA expression in 38 RA patients and 40 healthy individuals. Pearson’s Chi square test and two independent-samples Wilcoxon tests were used for statistical analysis.

**Results:**

Eight single nucleotide polymorphisms (SNPs) (rs5029937, rs3799491, rs598493, rs2307859, rs146534657, rs2230926, rs661561, and rs582757) were identified in PBMCs of RA patients. One new mutation (14284 T > A) was identified in synovial fluid mononuclear cells from one RA case. rs146534657 was identified for the first time in two RA cases. Patients with rs146534657 (12411 A > G, Asn102Ser) AG genotype or rs2230926 (12486 T > G, Phe127Cys) TG genotype had poor outcome. Significantly lower *A20* mRNA expression was found in PBMCs from RA patients compared with healthy individuals (*p* < 0.001). There was a higher *A20* mRNA expression in RA patients with rs2230926 TG genotype and rs146534657 AG genotype (11.56 ± 7.39) than patients with rs2230926 TT genotype and rs146534657 AA genotype (5.63 ± 4.37) (*p* = 0.031).

**Conclusion:**

Significantly lower *A20* expression was found in RA patients. The polymorphisms of *A20* were characterized in RA patients. We detected rs146534657 for the first time and identified a new *A20* mutation (14284 T > A). *A20* rs2230926 TG genotype and rs146534657 AG genotype may be related to poor outcome in RA patients.

**Electronic supplementary material:**

The online version of this article (doi:10.1186/s12967-015-0566-1) contains supplementary material, which is available to authorized users.

## Background

Rheumatoid arthritis (RA) is a systemic autoimmune inflammatory disease characterized by proliferative synovitis, the infiltration of inflammatory cells into synovial tissue, progressive joint destruction and disability. Several changes in the T cell compartment have been described in immune-mediated diseases [1]. Abnormal T cell immunity plays a critical role in the development of RA. Inflammatory mediators, such as interleukin-6 (IL-6), interleukin-1 (IL-1) and tumor necrosis factor alpha (TNF-α), are significantly over expressed in RA. Recently, RA treatment has been transformed by the development of biologics targeting TNF-α and IL-6 [2]. Moreover, many inflammatory mediators involved in the pathology of RA are regulated by nuclear factor kappa B (NF-κB) transcription factors [3]. Overexpression of NF-κB is a common characteristic of RA. Although the RA etiology is not fully understood, it is known that a strong genetic component plays a major role in this disease [4, 5].

A20, also known as TNFAIP3, was first identified as a TNF primary response transcript encoding a 790 amino acid protein with a unique zinc finger motif, and it is a ubiquitin-editing enzyme that is an essential negative regulator of inflammation via its zinc finger domains in C-terminus and OUT (ovarian tumor) domain in N-terminus. *A20* acts as a negative-feedback regulator of NF-κB activation in response to multiple stimuli, including TNF, IL-1, TLR (Toll-like receptor) and NLR [Nod (nucleotide-binding oligomerization domain)-like receptor] ligands [6, 7].

Genome-wide association studies have implicated the *A20* locus in susceptibility to multiple autoimmune diseases in different cohorts, including RA, systemic lupus erythematosus (SLE), psoriasis, celiac disease, type 1 diabetes, inflammatory bowel disease, and coronary artery disease. Alterations in the activity or expression of *A20* may influence the pathogenesis of RA [8–10]. Many *A20* single nucleotide polymorphisms (SNPs) were found to be associated with the susceptibility to autoimmune disease [11–13].

There are a number of studies regarding the susceptibility of *A20* SNPs in RA [11]. However, only a few of these studies have found an association between *A20* gene polymorphisms and the risk of RA in Chinese population [14]. Moreover, there are no data comparing the characteristics of *A20* polymorphisms and mutations in different fluids and tissues of RA. Thus, in this study, we analyzed the distribution of *A20* gene polymorphisms in peripheral blood and synovial fluid as well as the *A20* expression level, and evaluated the potential association of *A20* polymorphisms with clinical characteristics of RA in Chinese population.

## Methods

### Study population

This study included 50 cases with untreated RA (7 males and 43 females, age: 13–74 years, median age: 55.6 years), and 40 healthy individuals (6 males and 34 females, age: 18–70 years, median age: 55.0 years) served as controls. (Among them, DNA samples were isolated from 50 RA patients and 30 healthy for SNPs analysis, while RNA samples were isolated from 38 RA patients and 40 healthy individuals for A20 gene expression level detection). There was no significant difference in the distribution of the genders or ages between the cases and controls. RA diagnoses were based on the American College of Rheumatology criteria and expert opinion (1987 ACR criteria) [15]. All RA patients were assessed for clinical disease activity by a trained rheumatologist using disease activity score 28 (DAS 28), and their erythrocyte sedimentation rate (ESR), C reactive protein (CRP), rheumatoid factor (RF), and anti-cyclic citrullinated peptide antibody (CCP) were collected [16]. RF and CRP were measured by immune nephelometry, and anti-CCP antibody was measured by enzyme-linked immunoabsorbent assay (ELISA). In the active phase, all of the parameters were abnormal, including the ESR: 69.61 ± 34.63 mm/h, CRP: 35.67 ± 28.68 mg/l, RF: 247.89 ± 377.42 IU/ml, DAS28: 7.50 ± 1.28, and anti-CCP antibody positivity was found for 29 patients, while anti-CCP antibody negativity was found for 16 patients, anti-CCP antibody was not detected in 5 patients. The healthy individuals were in healthy status without any cancer, type 2 diabetes, hypertension, or autoimmune inflammatory disease. Neither RA patients nor healthy individuals smoked. Peripheral blood mononuclear cells (PBMCs) were isolated from heparinized venous blood by Ficoll-Paque gradient centrifugation. The synovial fluid was collected from RA patients whose knees were examined for therapeutic purpose. RNA and DNA extraction and cDNA synthesis from PBMCs were performed according to the manufacturer’s instructions (Trizol, Invitrogen, USA, and Superscript III Kit, Gibco, Gaithersburg, MD, USA) [17, 18]. All procedures were conducted according to the guidelines of the Medical Ethics Committee of the Health Bureau of the Guangdong Province in China.

### PCR and sequencing

To amplify different domains of genomic DNA that cover *A20* exons 2–9 (coding region) and exon/intron junctions of the *A20* gene according to the structure of the *A20* gene, 11 pairs of primers were purchased (Additional file 1: Table S1). PCR was performed as described in our previous study [19]. The PCR products were used for mutation analysis of the *A20* coding sequence by direct sequencing using the Big Dye Terminator v3.1 Cycle Sequencing Kit (Perkin Elmer, ABI) and the ABI PRISM 3100-Avant genetic analyzer. Direct sequencing was performed by Invitrogen Biotechnology Company. Sequences of different samples from patients with RA and healthy individuals were analyzed with BLAST software (http://blast.ncbi.nlm.nih.gov/Blast.cgi) to identify polymorphisms or mutations in the *A20* gene.

### Quantitative Real-time PCR (qRT-PCR)

To compare the *A20* mRNA expression level, qRT-PCR was performed using specific *A20* primers (Additional file 1: Table S1), and the relative amount of the genes of interest and the *β*_*2*_*M* reference gene was measured in two independent assays. Specific amplification of the PCR products was analyzed by melting curve analysis. The data were presented as the relative expression of the genes of interest compared with the internal control gene as determined by the $2(^{{ - \varDelta {\text{C}}_{\text{T}} }} )$ method. qRT-PCR was performed as previously described [20–22].

### Statistical analysis

All statistical analyses were performed with SPSS (v. 13.0) software. A P value of less than 0.05 was considered statistically significant. Pearson’s Chi square test was used to compare the distribution of genotypes and alleles between RA group and healthy control group. In cases in which the genotype had a frequency of less than 1, Fisher’s exact test was applied. Two independent-samples Wilcoxon tests were performed to compare the median *A20* mRNA expression level of RA group and controls group.

## Results

### The distribution and frequency of *A20* SNPs in RA

We used 11 primer pairs, which cover the *A20* coding region, and several introns to amplify segments of *A20* genomic DNA, and positive PCR products were confirmed by sequencing. DNA samples from 50 cases with RA and 30 healthy individuals were selected for this part of the study. We detected eight SNPs of *A20* gene: rs5029937 (position 11571 with a nucleotide substitution of G to T), rs3799491 (position 11812, G > A), rs598493 (position 11822, T > C), rs2307859 (position 12387–12389 delCCT), rs146534657 (position 12411, A > G, resulting in an amino acid substitution at position 102 from asparagine to serine, Asn 102 Ser), rs2230926 (position 12486, T > G, resulting in an amino acid substitution at position 127 from phenylalanine to cysteine, Phe127Cys), rs661561 (position 13751, A > C) and rs582757 (position 14244, C > T).

**Figure 1:**
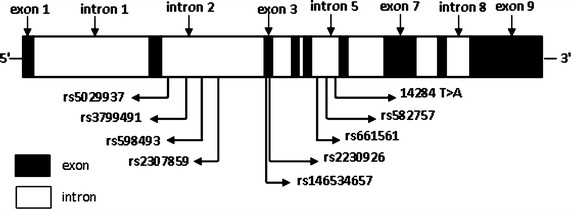
Location of SNPs in the *A20* gene locus.

**Figure 2:**
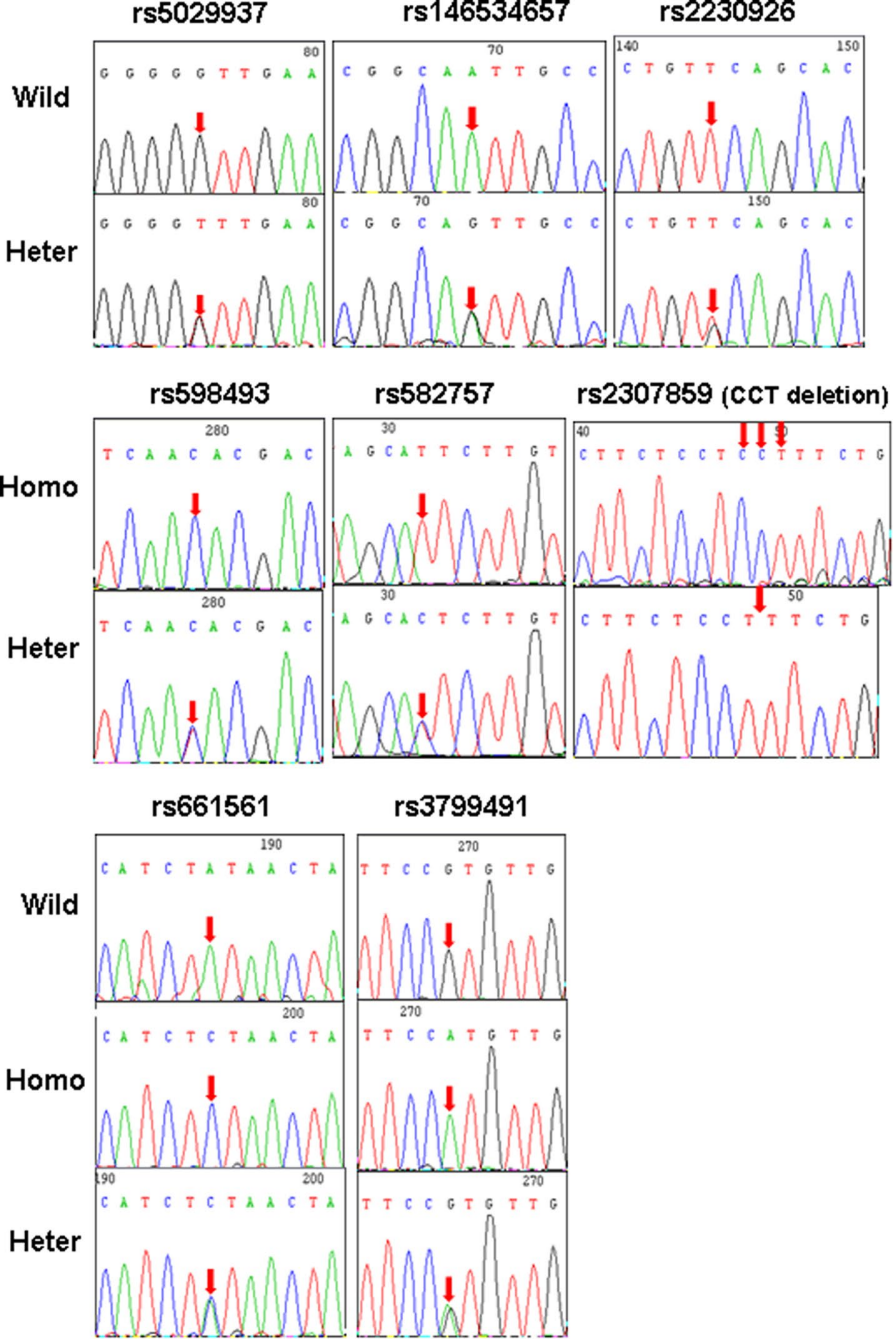
Identification of *A20* SNPs in RA patients. *Arrows* indicate sites of nucleotide changes. *Wild* wild-type, *Homo* homozygous, *Heter* heterozygous.

Most SNPs are located in intron 2 and 5, while two SNPs (rs146534657 and rs2230926) are located in exon 3 (Table 1; Figures 1, 2). However, the SNP frequencies appeared to be lower in the detected samples; the highest frequency was 24% (12/50 cases) for rs3799491 GA/AA genotype, and the lowest was 4.0% (2/50 case) for rs5029937 GT genotype, rs146534657 AG genotype, and without rs2307859 genotype respectively. In addition, the genotypes of four SNPs, rs2307859 (CCT deletion), rs661561 (homozygous), rs582757 (homozygous) and rs598493 (homozygous), appeared to be common genetic alterations in RA as they were identified in 41 cases, while the remaining 9 cases demonstrated different genotypic characteristics. However, 6 of the 8 SNPs were also identified in the healthy control group with similar frequency, and rs146534657 AG genotype and rs2307859 wild genotype were not identified in the healthy controls. However, there was no statistically significant difference in the proportion of genotypic data between the RA group and healthy group (Table 2). Moreover, genetic alterations in RA and healthy samples with the rs2230926 TG genotype and/or rs146534657 AG genotype were different from those lacking both SNPs (Table 3).

**Table 1:** Identified SNPs of the *A20* gene in PBMCs and synovial fluid from RA

SNP number	Chromosome position	Gene position	Major/minor	Function
rs5029937	137874014	Intron 2	G/T	–
rs3799491	137874255	Intron 2	G/A	–
rs598493	137874265	Intron 2	C/T	–
rs2307859	137874830	Intron 2	12387_12389 delCCT	–
rs146534657	137874854	Exon 3	A/G	Asn102Ser
rs2230926	137874929	Exon 3	T/G	Phe127Cys
rs661561	137876194	Intron 5	C/A	–
rs582757	137876687	Intron 5	T/C	–
NEW1 (14284)	137876727	Intron 5	T/A	–

**Table 2:** Frequencies of the alleles and genotypes of the *A20* polymorphisms in RA patients (case) and healthy individuals (control)

SNPs	Genotype frequency n (**%**)				Allele frequency n (**%**)	
	Major homozygote	Heterozygote	Minor homozygote		Major allele	Minor allele
rs5029937	GG	GT	TT		G	T
Case	48 (96.0)	2 (4.0)	0 (0)		98 (98.0)	2 (2.0)
Control	28 (93.3)	2 (6.7)	0 (0)		58 (96.7)	2 (3.3)
p-value	–	1.000			–	1.000
rs3799491	GG	GA	AA	GA/AA	G	A
Case	38 (76.0)	11 (22.0)	1 (2.0)	12 (24)	87 (87.0)	13 (13.0)
Control	21 (70.0)	9 (30.0)	0 (0)	9	51 (85.0)	9 (15.0)
p-value	–	0.454	1.000*	0.555	–	0.722
rs598493	CC	TC	TT		C	T
Case	44 (88.0)	6 (12.0)	0 (0)		94 (94.0)	6 (6.0)
Control	26 (86.7)	4 (13.3)	0 (0)		56 (93.3)	4 (6.7)
p-value	–	1.000			–	1.000
rs2307859	–/CCT	Wild type				
Case	48 (96.0)	2 (4.0)				
Control	30 (100)	0 (0)				
p-value	–	0.525*				
rs146534657	AA	AG	GG		A	G
Case	48 (96.0)	2 (4.0)	0 (0)		98 (98.0)	2 (2.0)
Control	30 (100)	0 (0)	0 (0)		60 (100)	0 (0)
p-value	–	0.525*			–	0.528*
rs2230926	TT	TG	GG		T	G
Case	47 (94.0)	3 (6.0)	0 (0)		97 (97.0)	3 (3.0)
Control	28 (93.3)	2 (6.7)	0 (0)		58 (96.7)	2 (3.3)
p-value	–	1.000			–	1.000
rs661561	CC	AC	AA	AC/AA	C	A
Case	41 (82.0)	8 (16.0)	1 (2.0)	9 (18)	90 (90.0)	10 (10.0)
Control	24 (80)	6 (20)	0 (0)	6 (20)	54 (90.0)	6 (10.0)
p-value	–	0.678	1.000*	0.824	–	1.000
rs582757	TT	CT	CC		T	C
Case	44 (88.0)	6 (12.0)	0 (0)		94 (94.0)	6 (6.0)
Control	26 (86.7)	4 (13.3)	0 (0)		56 (93.3)	4 (6.7)
p-value	–	1.000			–	1.000

*n* number of participants.

* Fisher’s exact p value.

**Table 3:** *A20* genetic alterations with rs230926 and rs146534657 in PBMCs of RA patients and healthy individuals

Cases	rs5029937	rs3799491	rs598493	rs2307859	rs146534657	rs2230926	rs661561	rs582757
PHL^a^	Wild	Wild	Homo	Wild	Heter	Heter	Wild	Homo
LXS^a^	Wild	Heter	Homo	Del	Heter	Wild	Heter	Homo
WYF^a^	Heter	Wild	Homo	Wild	Wild	*Heter*	Heter	Homo
LWL^a^	Heter	Wild	Homo	Del	Wild	Heter	Homo	Homo
HI1	Heter	Wild	Homo	Del	Wild	Heter	Heter	Homo
HI2	Heter	Heter	Homo	Del	Wild	Heter	Heter	Homo

*HI* healthy individuals, *Wild* wild-type, *Homo* homozygous, *Heter* heterozygous, *Del* deletion.

^a^RA cases.

### Characteristics of *A20* SNPs in synovial fluid from RA patients

**Figure 3:**
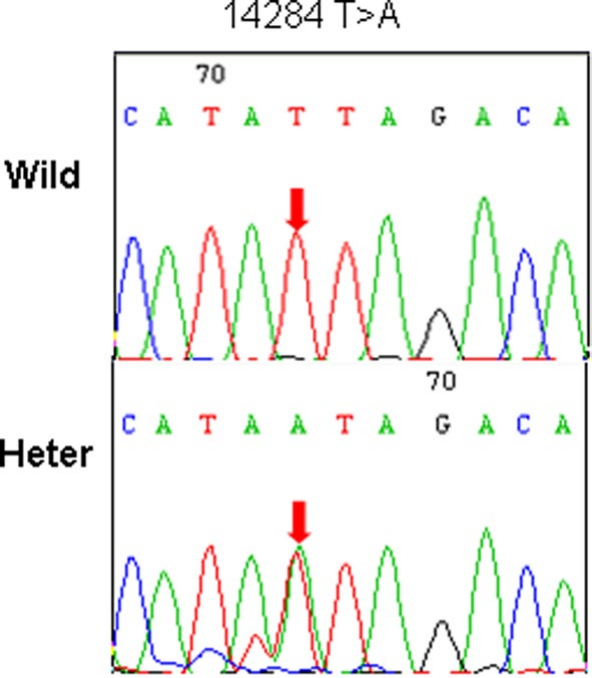
New mutation identified in *A20* in synovial fluid from a patient with RA. *Arrows* indicate sites of nucleotide changes. *Wild* wild-type, *Heter* heterozygous.

We further analyzed the characteristics of *A20* SNPs in cells from the synovial fluid and PBMCs from 11 cases with RA whose knees were examined for therapeutic purpose at the same time as peripheral blood collection. A total of seven *A20* SNPs (rs5029937, rs3799491, rs598493, rs2307859, rs2230926, rs661561 and rs582757) were identified. The identical SNPs in the synovial fluid and peripheral blood were identified in 10 RA cases (Table 4), and a new mutation (14284 T > A) was identified in synovial fluid from only one RA case whose peripheral blood sample did not contain this mutation (Table 4; Figure 3).

**Table 4:** *A20* gene polymorphism characteristics in peripheral blood and synovial fluid from RA patients

Case no.	Samples	rs5029937	rs3799491	rs598493	rs2307859	rs2230926	rs661561	rs582757	14284 T > A
1	S	Wild	Wild	Homo	Del	Wild	Homo	Homo	Wild
	B	Wild	Wild	Homo	Del	Wild	Homo	Homo	Wild
2	S	Wild	Wild	Homo	Del	Wild	Homo	Homo	Wild
	B	Wild	Wild	Homo	Del	Wild	Homo	Homo	Wild
3	S	Wild	Wild	Heter	Del	Wild	Heter	Heter	Wild
	B	Wild	Wild	Heter	Del	Wild	Heter	Heter	Wild
4	S	Wild	Wild	Homo	Del	Wild	Homo	Homo	Heter
	B	Wild	Wild	Homo	Del	Wild	Homo	Homo	Wild
5	S	Wild	Wild	Homo	Del	Wild	Homo	Homo	Wild
	B	Wild	Wild	Homo	Del	Wild	Homo	Homo	Wild
6	S	Heter	Wild	Homo	Wild	Heter	Heter	Homo	Wild
	B	Heter	Wild	Homo	Wild	Heter	Heter	Homo	Wild
7	S	Wild	Homo	Homo	Del	Wild	Homo	Homo	Wild
	B	Wild	Homo	Homo	Del	Wild	Homo	Homo	Wild
8	S	Wild	Wild	Homo	Del	Wild	Homo	Homo	Wild
	B	Wild	Wild	Homo	Del	Wild	Homo	Homo	Wild
9	S	Wild	Wild	Homo	Del	Wild	Homo	Homo	Wild
	B	Wild	Wild	Homo	Del	Wild	Homo	Homo	Wild
10	S	Wild	Heter	Homo	Del	Wild	Homo	Homo	Wild
	B	Wild	Heter	Homo	Del	Wild	Homo	Homo	Wild
11	S	Wild	Wild	Homo	Del	Wild	Homo	Homo	Wild
	B	Wild	Wild	Homo	Del	Wild	Homo	Homo	Wild

*B* peripheral blood, *S* synovial fluid, *Wild* wild-type, *Homo* homozygous, *Heter* heterozygous, *Del* deletion.

### The association between *A20* SNPs and *A20* expression level

**Figure 4:**
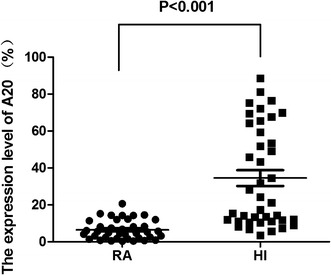
*A20* expression level in RA patients and healthy individuals (HI).

**Figure 5:**
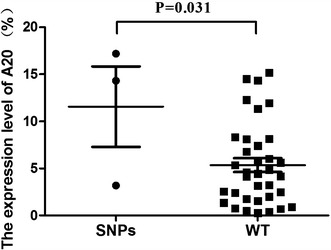
Comparison of *A20* expression level between SNPs (RA patients with rs2230926 TG genotype and rs146534657 AG genotype) and WT (RA patients with rs2230926 TT genotype and rs146534657 AA genotype).

**Figure 6:**
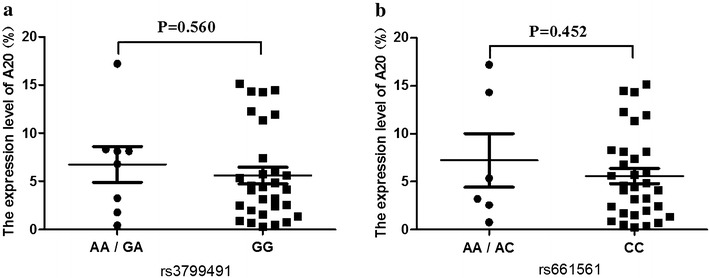
Comparison of the *A20* expression level between samples with rs3799491 or rs661561 SNPs. **a** *A20* expression level in patients with rs3799491 AA/GA genotypes and with rs3799491 GG genotype. **b** *A20* expression level in patients with rs661561 AA/AC genotypes (heterozygous) and with rs661561 CC genotype (homozygous).

We analyzed the *A20* expression level in cDNA samples from 38 RA patients and 40 healthy individuals by real-time PCR, and a significantly lower *A20* expression level (5.96 ± 4.82) was found compared with that in healthy individuals (34.54 ± 26.89) (p < 0.001) (Figure 4). We also compared the *A20* expression level of different SNPs, it appeared that there was a higher *A20* expression level in RA patients with rs2230926 TG genotype and rs146534657 AG genotype (11.56 ± 7.39) than patients with rs2230926 TT genotype and rs146534657 AA genotype (5.63 ± 4.37) (P = 0.031) (Figure 5). In addition, there was no significant difference of *A20* expression level between RA patients with rs3799491 AA/GA genotypes (6.75 ± 5.25) and with rs3799491 GG genotype (5.61 ± 4.79) (P = 0.560) (Figure 6a). The *A20* expression level in RA patients with rs661561 AA/AC genotypes (7.23 ± 6.82) appeared to be higher than with rs661561 CC genotype (5.59 ± 4.47); however, there was also no significant difference (p = 0.452) (Figure 6b).

### The association between *A20* alteration and RA clinical characteristics

First, we compared the difference of SNPs and the status in RA patients with or without anti-CCP antibody positive, we found there were no correlation between the eight SNPs and anti-CCP antibody. We also found that there was no significant difference of *A20* expression level between anti-CCP antibody positive and anti-CCP antibody negative RA groups (P > 0.05).

Among the 50 RA patients, rs2230926 TG genotype was identified in 3 RA patients, one patient both with rs2230926 TG genotype and rs146534657 AG genotype was poor response to immune suppression therapy, such as methotrexate, infliximab and anti-TNF-α antibody; the second and the third patients both with rs2230926 TG genotype and rs5029937 GT genotype suffered severe disease with systemic joint destruction. rs146534657 AG genotype was identified in two RA patients, the second patient suffered obvious joint deformity in multiple joints of the hands and the knee. Overall, the SNPs rs2230926 and/or rs146534657, which located in exon 3, appeared to be related to poor clinical outcome in RA. Moreover, there were no significantly different clinical characteristics for the patient with the newly detected mutation (14284 T > A).

## Discussion

*A20* plays a central role in the control of NF-kB activation, *A20* also negative regulates NLRP3 inflammasome to protect against arthritis [23]. It is known to be associated with susceptibility to multiple autoimmune diseases [6]. Polymorphisms and mutations in the *A20* gene are linked to various autoimmune diseases. Recent studies from different countries have reported that *A20* is frequently inactivated by deletions and/or mutations in autoimmune diseases, including RA [11]. Previous studies of *A20* in RA have demonstrated an association with several disease risk SNPs, including rs2230926, rs6920220 and rs10499194 [24]. However, the frequencies of the reported *A20* abnormalities in different reports are dissimilar. Moreover, our previous study suggested that the *A20* polymorphisms associated with disease susceptibility may be different for different ethnic groups [19]. Little is known about the genetic alteration characteristics of *A20* gene in Chinese population. In this study, we evaluated the role of the polymorphisms in the *A20* gene on the risk for RA.

A number of studies have demonstrated a strong association between rs2230926 and RA, and this SNP plays a functional role in the development of RA. rs2230926 is located in exon 3 of *A20*, and results in an amino acid (at 127 position) substitution from phenylalanine to cysteine (Phe127Cys). This risk allele (Cys127) leads to reduced inhibition of NF-kB activation or reduced *A20* mRNA levels [25]. There is a significant association between rs2230926 and increased risk for SLE and RA in the Japanese population [26]. Moreover, the rs2230926 polymorphism is associated with RA susceptibility in Europeans and Asians [24]. In this study, we found only three cases with rs2230926 TG genotype in 50 RA samples; however, we also found two cases with rs2230926 TG genotype among 30 healthy individuals. Therefore, this preliminary result may indicate a lower incidence of rs2230926 TG/GG genotype in Chinese RA patients, as least in our study group. Although patients with rs2230926 TG genotype demonstrated refractory and poorer outcome, there was no significant association between RA patients and healthy individuals, which was similar to findings in our previous study on T cell acute lymphoblastic leukemia (T-ALL) [19]. Analogous results were reported by Orozco et al. who found that rs2230926 is not independently associated with RA [27]. In addition, findings from a Korean group showed that rs2230926 in *A20* is not associated with RA susceptibility in the Korean population [28]. However, results from Zhang et al. showed that rs2230926 is associated with RA risk in the northern Chinese population [29]. Because the RA patients and healthy individuals in this study were all from southern China, whether there are different genotypes of *A20* gene in northern and southern Chinese people remains an open question. An alternative may be that, due to the limited samples in this study, the association between rs2230926 and RA in Chinese population requires further investigation with a greater number of samples.

Interestingly, we found two RA patients with rs2230926 TG genotype did not accompany rs2307859 (CCT deletion) which was a common alteration in 48 RA patients and 30 healthy individuals, it may be possible that absence of rs2307859 genotype is a risk factor to RA when rs2230926 TG genotype is present. Moreover, the genetic alteration of *A20* in one patient with both rs2230926 TG genotype and rs146534657 AG genotype and poor outcome appeared to be different from most RA patients because it appeared with rs661561 AA genotype, which was found in almost all of the RA patients and healthy individuals with homozygous (CC genotype) or heterozygous (AC genotype) [30]. Whether such genetic alterations in *A20* leads to high risk for RA needs further investigation. In addition, similar genetic alterations in *A20* were identified in T-ALL [19].

In this study, we detected rs146534657 for the first time in two RA patients. This SNP which was described in GenBank but not identified for any disease, is a nonsynonymous variant located in exon 3 of *A20* and results in an amino acid (at position 102) substitution from asparagine to serine (Asn 102 Ser). According to the clinical information of the patients, those who had both rs2230926 TG genotype and rs146534657 AG genotype had poor outcome, and whether rs2230926 and rs146534657 are associated with refractory RA requires further investigation with more samples to demonstrate.

In this study, we also analyzed the alteration of SNPs in *A20* that were reported in different autoimmune diseases, such as rs5029937, which was strongly associated with RA in a large European RA cohort [27, 31] and significantly associated with SLE in southwestern Chinese population [32]; rs582757, which was reported to be associated with rheumatic heart disease (RHD) in the Chinese Han population [33] and weakly associated with reduced risk for RA in Caucasians [34], also associated with psoriasis [35]; rs598493, which was significantly increased in the GG genotype in Graves’ disease (GD) [30]; and rs3799491, which has not been reported in RA or other diseases. However, in this study, rs5029937 GT genotype was identified only in two RA patients who with rs2230926 TG genotype and suffered severe disease with systemic joint destruction, and whether rs5029937 is associated with the destruction of bone and cartilage in RA need further investigation. Moreover, rs582757 and rs598493 were identified in all samples including RA patients and healthy individuals as having a homozygous or heterozygous genotype; thus, it could be concluded that these are a common genotype in the Chinese population. In addition, rs3799491 GA/AA genotype was found in 12 out of 50 RA patients and 9 out of 30 healthy individuals, and we found no significant difference in these alterations between RA patients and healthy individuals. These results may indicate that the *A20* polymorphisms associated with RA susceptibility are different in different populations.

To define the *A20* gene polymorphism characteristics of synovial fluid from RA patients, we compared *A20* polymorphisms in samples from peripheral blood and synovial fluid from 11 RA patients. Interestingly, one new mutation (14284 T > A) was identified in synovial fluid from one RA patient. This result may indicate the possibility of inconsistent genetic alterations in different tissues from the same patient. However, the significance and frequency of this new mutation need further investigation.

As a NF-kB negative regulator, A20 is critical for limiting inflammation by terminating TNF-induced NF-kB responses in vivo [36]. We also analyzed the *A20* expression level in PBMCs of RA patients, and a significantly lower *A20* expression level may be related to enhanced NF-kB and RA pathogenesis [22, 37]. There was a negative relationship between the *A20* mRNA expression level and RA activity. Interestingly, PBMCs from RA patients with the rs2230926 TG genotype and rs146534657 AG genotype had a higher *A20* expression level compared with RA patients with rs2230926 TT genotype and rs146534657 AA genotype, and a similar finding was reported for T-ALL in our previous study [19]. This result may imply that such a rs2230926 TG genotype may be related to the maintenance of *A20* expression level. However, this hypothesis is based only on results from a limited case analysis, and further research involving more samples is needed to determine representative results. Moreover, this finding appeared to be inconsistent with the disease status of patients; thus, remaining an open question.

## Conclusion

In conclusion, we characterized the *A20* gene polymorphisms of RA, detected the SNP rs146534657 for the first time in RA, and found a new mutation (14284 T > A) in synovial fluid of RA. Our data suggested that the rs2230926 TG genotype and rs146534657 AG genotype might be related to RA poor outcome. Significantly lower *A20* mRNA expression was found in RA patients. However, further research involving more samples is needed to determine representative results.

## Additional files

Additional file 1:
**Table S1.** Details of primers used in the PCR and real-time PCR.
