# Supplementary material for: Characteristics of A20 gene polymorphisms and clinical significance in patients with rheumatoid arthritis
Source: J Transl Med. 2015 Jul 5;13:215. doi: 10.1186/s12967-015-0566-1 (PMC4491428; doi:10.1186/s12967-015-0566-1)
Supplement: Additional file 1: — Table S1. Details of primers used in the PCR and real-time PCR. [file 12967_2015_566_MOESM1_ESM.doc]

Supplemental table 1 Details of primers used in the PCR and real-time PCR

| primers | Sequence | Function |
| --- | --- | --- |
| A20exon2-f | 5’- ggagtcgtattaaagtcaggctaa | PCR for part of exon 2 amplification |
| A20exon2-r | 5’- ggcaaaagaaacacaacagaac |
| A20exon3-f | 5’- ttgctgggtcttacatgcag | PCR for part of exon 3 amplification |
| A20exon3-r | 5’- cccaccatggagctctgtta |
| A20exon4-f | 5’- gggagtacaggatacattcaagc | PCR for part of exon 4 amplification |
| A20exon4-r | 5’- gctgaaagcatttaagtacagatcc |
| A20exon5-f | 5’- acctaagggcctcattttcc | PCR for part of exon 5 amplification |
| A20exon5-r | 5’- agcaaaaaggaaaaccctga |
| A20exon6-f | 5’- tgagatctacttacctatggccttg | PCR for part of exon 6 amplification |
| A20exon6-r | 5’- cagatgacacaggagagagctg |
| A20exon7-1-f | 5’- ggttctacaattcttgccataatcc | PCR for part of exon 7 amplification |
| A20exon7-1-r | 5’- caagtgccttgtgtggtctg |
| A20exon7-2-f | 5’- cacaacggattttgtgaacg | PCR for part of exon 7 amplification |
| A20exon7-2-r | 5’- aggaacaaaaccccttctgg |
| A20exon8-f | 5’- ctctgtatcggtggggtgac | PCR for exon 8 amplification |
| A20exon8-r | 5’- caaaaagcatcgaacacacg |
| A20exon9-f | 5’- tgatctgcctgttctttcca | PCR for exon 9 amplification |
| A20exon9-r | 5’- gggttcagaggatagcacca |
| A20-10-f | 5’- ttattttaatagcctcatgtggaata | PCR for rs582757 amplification |
| A20-10-r | 5’-aggttagttgacctctctgagct |
| A20-11-f | 5’-cacttgccaaaggagattaagg | PCR for rs5029937 amplification |
| A20-11-r | 5’-tgaccattccactttagccttt |
| A20-for | 5′-ctgggaccatggcacaactc | Real-time RT-PCR for A20 expression level analysis |
| A20-rev | 5'-cggaaggttccatgggattc |
| NF-κB-for | 5′-ccacaagacagaagctgaag | Real-time RT-PCR for NF-κB expression level analysis |
| NF-κB-rev | 5′-agatactatctgtaagtgaacc |
| β2m-for | 5'-tacactgaattcacccccac | Real-time RT-PCR for β2m amplification as reference |
| β2m-rev | 5'-gcggcatcttcaaacctc |
